# Tumor-localized immunomodulation: a critical advance in engineering CAR-t cells for solid malignancies

**DOI:** 10.1097/MS9.0000000000004256

**Published:** 2025-11-04

**Authors:** Maryam Abid, Ursula Abu Nahla, Muhammad Nabeel Saddique

**Affiliations:** a Department of Radiotherapy and Medical Oncology, King Edward Medical University, Lahore, Pakistan; b Faculty of Medicine, Hebron University, Hebron, Palestinian Territories

**Keywords:** CAR-T cells, checkpoint inhibitors, immunotherapy, solid tumors, tumor microenvironment

## Abstract

Chimeric antigen receptor (CAR)-T cell therapy, despite revolutionizing hematological malignancies, remains limited in solid tumors due to immunosuppressive microenvironments and systemic toxicities from combination immunotherapies. Recent engineering innovations demonstrate that physically linking anti-PD-L1 antibodies to interleukin-12 within CAR-T cells creates tumor-localized immunomodulation, concentrating therapeutic activity at PD-L1-positive sites while minimizing systemic exposure. In preclinical models, PD-L1-binding IL-12 fusion proteins achieved superior antitumor responses (100 vs. 50% complete responses) compared to non-binding controls, with significantly reduced inflammatory toxicity. Spatial proteomic analysis revealed comprehensive tumor microenvironment remodeling including enhanced CD8+ T cell infiltration and reduced immunosuppressive myeloid populations. Validation in human CAR-T cells targeting TAG72-positive ovarian cancer confirmed appropriate PD-L1 binding and enhanced cytotoxicity. This rational engineering strategy addresses multiple barriers simultaneously through molecular sequestration, offering a promising platform applicable to alternative checkpoint-cytokine combinations and other cellular therapeutics. Clinical translation represents a critical next step for extending CAR-T efficacy to solid malignancies.

*Dear Editor,*

Chimeric antigen receptor (CAR)-T cell therapy represents a watershed achievement in cancer immunotherapy, yet its remarkable success in hematological malignancies has not readily translated to solid tumors. Recent advances in engineering strategies now promise to bridge this therapeutic gap. A pivotal study demonstrates that physically linking checkpoint blockade to localized cytokine delivery at tumor sites substantially improves efficacy while minimizing systemic toxicity – a critical distinction for advancing these powerful therapies into clinical practice^[1]^.

The immunosuppressive tumor microenvironment (TME) of solid malignancies presents formidable barriers including poor T cell infiltration, rapid exhaustion, and inadequate engagement of endogenous immunity^[2]^. While immune checkpoint inhibitors and pro-inflammatory cytokines show individual promise, their systemic combination produces severe dose-limiting toxicities. Murad and colleagues addressed this challenge through an elegant molecular engineering strategy: they engineered CAR-T cells to secrete bifunctional fusion proteins that physically link anti-PD-L1 antibodies to interleukin-12 (IL-12). This design concentrates therapeutic activity at PD-L1-positive tumor sites while minimizing systemic exposure.

Using spatial proteomic analysis, the researchers demonstrated comprehensive TME remodeling in prostate and ovarian cancer models. CAR-T cells secreting PD-L1-binding IL-12 fusion proteins achieved 100% curative responses in prostate tumors compared to 50% for non-binding controls, while showing substantially reduced systemic inflammation. Mechanistically, these engineered cells enhanced CD8+ T cell infiltration, increased activation markers, and reduced immunosuppressive myeloid populations. Critically, tumor-anchored IL-12 outperformed CAR-T cells combined with systemically administered IL-12 and anti-PD-L1 antibody, even at reduced IL-12 doses, demonstrating the therapeutic value of molecular sequestration beyond simple CAR-mediated delivery.

The validation in human systems strengthens translational prospects. Engineered human CAR-T cells targeting TAG72-positive ovarian cancer cells showed appropriate PD-L1 binding, enhanced tumor cytotoxicity, and increased interferon-gamma production *in vitro*, laying essential groundwork for clinical evaluation^[1]^.

This platform approach has broader applicability across multiple dimensions. The bifunctional design strategy could extend to other CAR targets, alternative checkpoint-cytokine combinations, or alternative cellular therapeutics including CAR-NK cells or tumor-infiltrating lymphocytes. The sequestration principle is particularly valuable for cytokines with narrow therapeutic windows (IL-2, IL-18, and TNFα) where systemic administration remains prohibitively toxic^[3]^. Moreover, the design framework accommodates advanced engineering features such as inducible expression systems or logic-gated circuits for refined spatiotemporal control.

However, several questions warrant further investigation. First, performance in truly “immunologically cold” human tumors with minimal baseline immune infiltration requires characterization. Second, the durability of responses beyond observation periods, and whether long-term control reflects CAR-T cell persistence, epitope spreading, or established immune memory, needs clarification. Third, the disappointing performance of TGFβ-trapping fusions despite supportive preclinical literature suggests that mechanistic insights about cytokine and checkpoint combinations remain incomplete and may prove tumor-context dependent.

The study also does not extensively address resistance mechanisms, particularly antigen loss – a persistent challenge in solid tumor CAR-T therapy. Whether localized IL-12 can overcome this through enhanced epitope spreading or recruitment of endogenous immunity remains to be established. Notably, the increased expression of alternative checkpoint molecules (VISTA, TIM-3, and LAG-3) in treated tumors suggests potential resistance pathways that may require further engineering solutions.

Despite these open questions, this work represents a paradigm shift in rational design of “armored” CAR-T cells capable of functioning effectively in hostile tumor environments. The comparison of PD-L1-binding versus non-binding IL-12 fusions provides compelling evidence for the therapeutic value of tumor localization beyond simple CAR-mediated targeting. For clinical translation, this distinction is particularly important; dose-limiting toxicities have repeatedly derailed promising immunotherapy combinations, making safety-enhanced designs essential for regulatory approval and patient tolerability^[4]^.

As the field grapples with extending CAR-T efficacy to solid tumors – which comprise approximately 93% of all malignancies – approaches that simultaneously address multiple barriers through elegant molecular engineering merit serious attention. The next critical steps involve demonstrating safety and efficacy in early-phase clinical trials and identifying which patient populations and tumor types are most likely to benefit from this innovative strategy. This manuscript was prepared in compliance with Transparency in the Reporting of Artificial Intelligence – The TITAN Guideline 2025^[5]^.

## Data Availability

Not applicable.
